# Entropy-Driven Intelligent Diagnosis for SMR Loss of Coolant Accidents: A CNN-LSTM-Attention Hybrid Model for Break Size Assessment

**DOI:** 10.3390/e28070745

**Published:** 2026-07-01

**Authors:** Lang Yang, Jichong Lei

**Affiliations:** 1 School of Humanity, Shanghai University of Finance and Economics, Shanghai 200433, China; hnityl@126.com; 2 School of Marxism, Hunan Institute of Technology, Hengyang 421002, China; 3 School of Safety and Management Engineering, Hunan Institute of Technology, Hengyang 421002, China

**Keywords:** small modular reactor (SMR), loss of coolant accident (LOCA), break size assessment, entropy-driven diagnosis, CNN-LSTM-Attention, deep learning, spatiotemporal feature learning

## Abstract

Accurate break size assessment is critical for the safety response of small modular reactors (SMRs) during loss-of-coolant accidents (LOCAs). Traditional methods struggle with the rapid transient features, strong spatiotemporal coupling, and complex uncertainty characteristics of SMR-LOCA, leading to low accuracy and poor stability. To address these issues, this study proposes an entropy-driven intelligent diagnosis approach based on a CNN-LSTM-Attention hybrid model. The framework adopts information entropy for data uncertainty quantification, adaptive weighting, and loss constraint, so as to realize high-precision break size assessment. A time-series dataset covering break sizes from 0.05 to 10 cm^2^ was constructed using the PCTRAN/SMART platform. The CNN module extracts spatial coupling features of multi-sensor parameters, the LSTM module captures long-term temporal dependencies, and the attention mechanism dynamically weights key information to enhance feature representation under high uncertainty. Experimental results show that the model achieves a mean absolute error (MAE) of 0.096311, reducing errors by over 64.4% compared with baseline models; more than 90% of prediction errors are within ±5%, and the correlation coefficient reaches 0.994902. Based on the well-validated PCTRAN/SMART simulation platform, the proposed entropy-informed spatiotemporal learning framework provides a technical solution for intelligent LOCA diagnosis, uncertainty quantification, and safety assessment of SMRs.

## 1. Introduction

Small Modular Reactors (SMRs) represent a key direction in nuclear energy innovation [1]. With their modular design, inherent safety advantages, and flexible deployment capabilities, they have become a critical component of future energy systems. However, SMRs face multiple safety risks, and Loss of Coolant Accidents (LOCA), as the core design-basis accident, pose particularly significant risks [2]. Compared to traditional large reactors, SMRs significantly reduce the risk of LOCA occurrence through features such as passive safety systems and integrated design [3]. However, the faster pressure drop rates and more complex two-phase flow characteristics resulting from their compact design give the LOCA evolution process unique characteristics, presenting new challenges for breach size assessment and creating an urgent need for evaluation methods tailored to these characteristics [4]. Traditional LOCA assessment methods fall into three categories: physical modeling, expert systems, and statistical learning. Among these, physical modeling offers high accuracy but is computationally intensive; expert systems rely on knowledge bases and have limited adaptability; and while statistical learning improves efficiency, it struggles to handle complex nonlinear temporal relationships [5]. None of these methods can fully meet the practical demands of SMR-LOCA assessment. The development of deep learning technologies has opened new avenues for nuclear safety assessment. Convolutional neural networks (CNNs) can effectively extract spatially coupled features from multi-sensor data in nuclear power plants [6], and multi-scale CNN architectures are better able to simultaneously capture local and global features, thereby improving the accuracy of anomaly detection [7]; Long Short-Term Memory (LSTM) networks, with their superior temporal modeling capabilities, have been successfully applied to transient identification and early LOCA diagnosis in nuclear power plants, enabling rapid identification of accident types and preliminary estimation of breach size ranges [8]; the attention mechanism, by dynamically allocating weights to focus on critical time points and core parameters during the accident process, effectively improves the accuracy of feature representation for fault diagnosis and LOCA assessment [9]. Building on this, the CNN-LSTM-Attention hybrid architecture integrates the strengths of all three, forming a triple mechanism of “spatial feature extraction-temporal evolution modeling-key information focusing.” This architecture can simultaneously process spatiotemporal information while possessing strong robustness and feature adaptability [10], making it the preferred solution for the quantitative assessment of LOCA break sizes. Existing research has proposed several end-to-end evaluation frameworks based on this architecture; some frameworks have incorporated multi-task learning mechanisms to further enhance evaluation performance, achieving significant improvements in evaluation accuracy on SMR simulation data [11]. The construction of high-quality datasets is fundamental to the practical application of deep learning models. Since actual SMR-LOCA data is difficult to obtain, researchers primarily generate training data through numerical simulations. Relevant studies have completed adaptation and optimization for thermal-hydraulic codes such as RELAP5-3D and TRACE and have established comprehensive data generation frameworks covering multiple breach locations, sizes, and initial conditions, providing robust support for model training [12]. Most existing intelligent diagnosis methods for SMR-LOCA mainly adopt standard CNN, LSTM, or CNN-LSTM-Attention hybrid architectures. These methods focus on mining spatial and temporal correlations of sensor data, but they have three common limitations. First, most studies ignore the strong random uncertainty of transient parameters during LOCA evolution and fail to quantify and suppress noise interference. Second, many models only achieve satisfactory performance for medium and large break accidents, while their detection accuracy for small-break weak signals is poor. Third, a large number of existing models adopt fixed weight allocation rules, leading to insufficient adaptability under diverse accident working conditions. Different from the above literature, this work innovatively integrates an entropy-driven mechanism into the spatiotemporal learning framework. On the basis of retaining the strengths of CNN in spatial feature extraction and LSTM in temporal dependency capture, we use information entropy to quantify data uncertainty, construct adaptive feature weights combined with the attention mechanism, and realize a full-range high-precision assessment covering small, medium, and large break sizes. This is a targeted improvement over the deficiencies of previous SMR-LOCA diagnosis models. Currently, while some progress has been made in SMR-LOCA break size assessment based on the CNN-LSTM-Attention architecture, most existing models cannot effectively cope with data uncertainty and small-break detection challenges. In view of this, this paper carries out customized modeling for the SMART reactor and realizes a full-range high-precision evaluation. The research results can provide important technical support for the intelligent diagnosis of LOCA accidents in SMRs.

## 2. Software and Methods

### 2.1. Introduction to SMART Reactor

**Figure 1:**
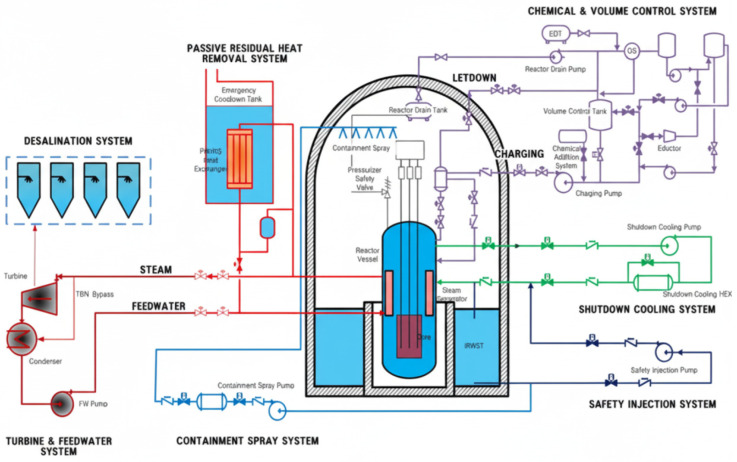
SMART System Flowchart.

SMART is an advanced small modular pressurized water reactor (SMR) developed under the leadership of the Korea Atomic Energy Research Institute (KAERI) [13]. Its latest version, SMART100, received standard design approval from the Korea Nuclear Safety and Security Commission in September 2024. Designed to enhance safety, economic efficiency, and versatility, it has a thermal power of 330 MWth and an electrical output of approximately 100 Mwe (Figure 1). With a design life of 60 years and a refueling cycle of three years, it significantly outlasts traditional large reactors. This reactor type features an integrated primary system design. The core assemblies consist of standard PWR low-enriched uranium dioxide fuel assemblies that have been optimized for miniaturization. Additionally, the core, eight spiral-tube steam generators, one pressurizer, four coolant pumps, and 25 control rod drive mechanisms are integrated within a single reactor pressure vessel. The built-in equipment design eliminates the risk of a LOCA caused by main steam pipe rupture and reduces the number of primary circuit openings and potential leakage points; Its cooling system is significantly simplified based on the typical PWR thermal cycle. The primary coolant circulates between the core and the steam generators, driven by an internal main pump. The secondary system is similar to that of a conventional PWR but scaled down to match the reduced electrical output. The integrated design eliminates traditional main pipelines, thereby eliminating the risk of a major water loss accident at its source. SMART’s safety strategy adheres to the principle of defense in depth. By combining inherent safety features such as low power density and a negative temperature coefficient with highly reliable passive safety systems, including a passive residual heat removal system, it ensures that the reactor can maintain a safe state for at least 36 h during an accident without requiring AC power or operator intervention. Preliminary safety analyses have also verified its robust response capabilities and design reliability against various design basis accidents.

### 2.2. Introduction to PCTRAN Simulation Platform

**Figure 2:**
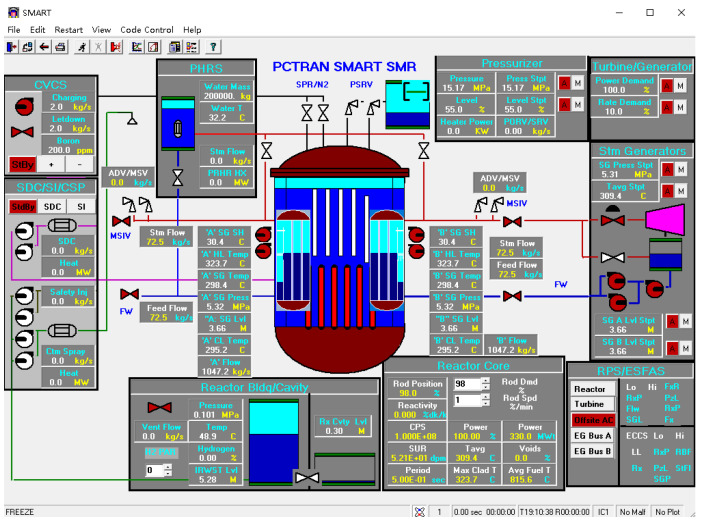
PCTRAN/SMART Graphical User Interface.

PCTRAN is a real-time simulation software developed by Micro-Simulation Technology (MST) in the United States, mainly used for nuclear power plant safety analysis and operator training [14]. It has been widely used in the global nuclear industry and regulatory agencies and includes models of various nuclear power plants such as AP1000, SMART, and NuScale [15]. The PCTRAN/SMART simulation platform (Figure 2) used in this study strictly follows the standard requirements of Korean APR1400 and OPR1000 units, and its model has been certified and used by five institutions, including the Korea Nuclear Safety Research Institute [16]. The platform can simulate the normal operation and various accident conditions of SMRs, providing real-time feedback on operating parameters such as temperature, pressure, and flow rate. The thermal-hydraulic calculation module and transient parameter solution algorithm of PCTRAN/SMART have been fully benchmarked against validated thermal-hydraulic codes and actual transient data of pressurized water reactors. Key transient parameters, including system pressure, core coolant flow rate, primary coolant inventory, and the response characteristics of the Emergency Core Cooling System (ECCS) under LOCA conditions, have been verified to ensure the reliability of simulation outputs, which meet the requirements of nuclear safety transient analysis [17].

### 2.3. Dataset Construction

To construct a high-quality SMR-LOCA breach dataset, this paper conducts systematic data generation for the SMART reactor type using the PCTRAN/SMR simulation platform [18,19]. By meticulously designing simulation scenarios, controlling parameter variables, and expanding sample diversity, the dataset is ensured to accurately reflect the dynamic characteristics of accidents involving different breaches, thereby providing support for model training and validation. The break size design employs concentration gradient sampling. Combined with the industry classification standard for Loss of Coolant Accidents (LOCA) in pressurized water reactors and the design characteristics of the SMART reactor, the break size range of 0.05–10 cm^2^ is selected to fully cover small, medium, and large LOCA working conditions of SMRs. The range is segmented into 200 gradient levels with 0.05 cm^2^ intervals, covering the three typical SMART-LOCA accident scenarios at an average step size. The 0.05 cm^2^ sampling interval is set to capture the characteristic signals of tiny break leakage, and this approach achieves “gap-free” coverage of the 0.05–10 cm^2^ range, ensuring no sensitive intervals are omitted, and each size corresponds to an independent simulation case, guaranteeing data independence and completeness. Simulation parameters strictly align with SMR operational characteristics, using a SMART unit rated at 300 MWe, a primary loop design pressure of 15.5 MPa, and passive safety systems as the baseline. Each simulation lasts 300 s with a sampling frequency of 1 Hz, generating 300-step time-series data containing 93 key monitoring parameters. The dataset is segmented using a time-series partitioning strategy to prevent data leakage. All data is normalized using Z-scores and stored in CSV format, forming a comprehensive dataset system that establishes a solid data foundation for subsequent model training and validation.

There are obvious differences between normal steady-state operation and LOCA transient conditions of SMRs. Under steady-state conditions, all operating parameters such as pressure, flow rate, and temperature remain stable within the design range; while under LOCA transient conditions, rapid pressure drop, sharp changes of coolant flow, and two-phase flow phenomena occur continuously, showing strong time-varying characteristics. According to the break area, LOCA is divided into small LOCA, medium LOCA, and large LOCA, and different types of LOCA present completely different transient evolution rules of monitoring parameters.

**Figure 3:**
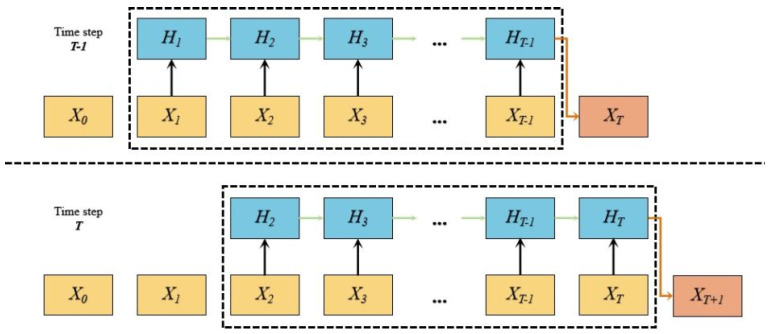
Schematic of the Sequence-to-One Prediction Model.

This research addresses the challenge of multivariate time series prediction, a task where conventional methodologies tend to decompose the multi-dimensional sequence into individual univariate prediction subtasks for separate processing (Figure 3). Such a fragmented approach, however, comes with notable drawbacks: it fails to capture the inherent intercorrelations across different variables, which in turn restricts the representational power of the established models [20]. To mitigate this limitation, we adopt a sequential input strategy in this work, wherein all physical parameters are fed collectively into a single neural network for unified training and prediction. In the prediction process, the historical sequence data vectors at each time step are input into the neural network in a chronological manner. The model then extracts the hidden state corresponding to the final time step, which is utilized to encode the complete input sequence. As shown in Figure 3, when the historical time step is set to *T* − 1, the input sample is defined as *X*_1:*T*−1_ = [*X*_1_, *X*_2_, …, *X_T_*_−1_], a six-dimensional sequence with a length of *T* − 1. The output *X_T_* is a six-dimensional vector that denotes the result of single-step prediction. The sample *X*_1:*T*−1_ is fed into the recurrent neural network at distinct time points with different sampling frequencies, generating a series of hidden states *H*_1_, *H*_2_, …, *H_T_*_−1_ that correspond to each time moment. Eventually, *H_T_*_−1_ is taken as the feature representation of the entire sequence and processed through a fully connected layer to yield the final prediction result *X_T_*. A key merit of this sequential input method is its ability to accept input sequences of arbitrary lengths without any modifications to the underlying model architecture. This flexibility allows the length of the historical time step to be treated as a tunable hyperparameter, and it is this very approach that we apply for the modeling and prediction tasks in the present study.

## 3. Theoretical Basis and Technical Platform

### 3.1. Convolutional Neural Network (CNN)

**Figure 4:**
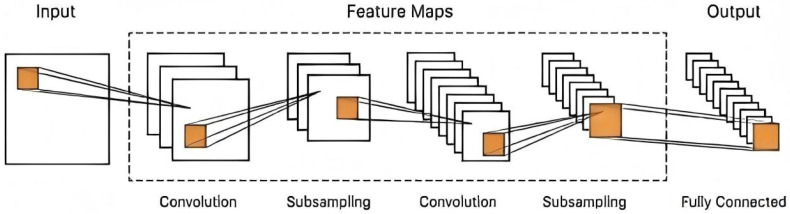
Basic Structure Diagram of a CNN Network.

CNN is a type of deep neural network that is particularly suitable for extracting spatial features from high-dimensional data [21]. Its basic structure includes an input layer, convolutional layers, pooling layers, and fully connected layers (Figure 4). The convolutional layer uses convolutional kernels to perform sliding convolution operations on the input data, extracting local spatial features; the pooling layer reduces the feature dimension through downsampling, improving the model’s robustness and computational efficiency; the fully connected layer maps the extracted features to the output space to complete classification or regression tasks [22]. In this study, the CNN layer is used to extract the spatial coupling features of SMR operation parameters, such as the correlation between primary loop pressure and pressurizer water level, and the coupling relationship between coolant flow rate and temperature.

### 3.2. Long Short-Term Memory (LSTM) Network

**Figure 5:**
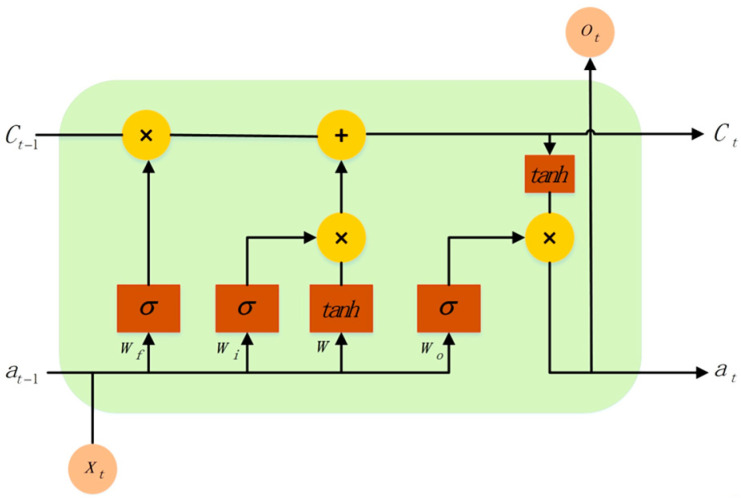
LSTM Cell Structure Diagram.

LSTM (Figure 5) is a special type of Recurrent Neural Network (RNN) that is suitable for processing time-series data and capturing long-term dependencies [23]. Its core structure includes input gates, forget gates, output gates, and memory cells. The input gate controls the addition of new information to the memory cell; the forget gate decides which information to discard from the previous memory cell; the output gate determines the output value based on the state of the memory cell; the memory cell is responsible for storing and transmitting long-term information, effectively solving the problems of gradient disappearance and gradient explosion in traditional RNNs [24]. In this study, the LSTM layer is used to capture the temporal dynamic evolution law of parameters in LOCA accidents, such as the time-varying trend of pressure drop and the dynamic change process of coolant flow rate.

### 3.3. Attention Mechanism

**Figure 6:**
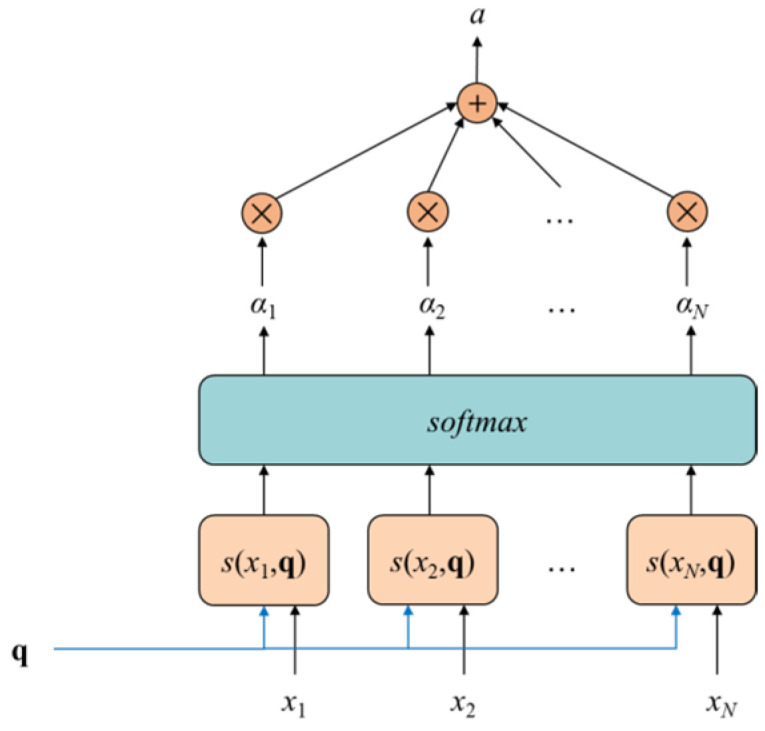
Schematic Diagram of Attention Weight Calculation Process.

The attention mechanism (Figure 6) simulates the selective attention process of the human brain, which can adaptively assign weights to different parts of the input data according to the importance of information [25]. Its basic calculation process includes determining query vectors (Query), key vectors (Key), and value vectors (Value); calculating the correlation score between the query vector and each key vector; normalizing the correlation score using the softmax function to obtain attention weights; weighting and summing the value vectors according to the attention weights to obtain the final attention output [26]. Common types of attention mechanisms include self-attention mechanism, cross-attention mechanism, and multi-head attention mechanism [27]. In this study, the self-attention mechanism is adopted to dynamically adjust the weights of different time steps and parameters in the time-series data, enhancing the model’s focus on key features related to break size.

The input of the self-attention layer is the fused spatiotemporal feature sequence, with a standard input tensor shape of R^B×T×F^. Where B is Batch size, representing the number of samples input in a single training iteration; T is Historical time step of time series, i.e., the length of continuous sampling points used for prediction; F is Number of feature dimensions, equal to 93 monitoring parameters collected in this SMR LOCA dataset.

Three independent trainable linear projection layers are used to map the input feature matrix *X* to generate Query (*Q*), Key (*K*), and Value (*V*) matrices, respectively. The calculation formulas are:

(1) $$Q=XW_{Q},K=XW_{K},V=XW_{V}$$

Where *W_Q_*, *W_K_* and *W_V_* denote learnable weight matrices for three projection layers, which are continuously optimized during model training. The dimensions of *Q*, *K*, and *V* are consistent with the input feature dimension R^B×T×F^.

### 3.4. Entropy-Driven Calculation and Optimization Strategy

The entropy-driven design is the core innovation of this study, which is used to quantify the uncertainty of multi-sensor time-series data under SMR LOCA conditions, optimize feature weights, and constrain model training. This section provides the complete mathematical formulas, weighting rules, and entropy-constrained loss function.

Information entropy is adopted to measure the uncertainty of each monitoring parameter sequence. A higher entropy value means the data contains more noise and less valid accident information. For a single time-series feature *X* = [*X*_1_, *X*_2_, …, *X_N_*], the Shannon information entropy is defined as:

(2) $$H\left(x\right)=-\sum_{i=1}^{N}p\left(x_{i}\right)\log_{2}p\left(x_{i}\right)$$

Where *N* is total number of sampling points of a single parameter; *x_i_* is observed value at the *i*-th sampling moment; *p*(*x_i_*) is probability density of *x_i_* obtained by statistical distribution of time-series data; *H*(*x*) is information entropy of the corresponding monitoring parameter. For the 93-dimensional fused monitoring data in this work, we calculate the entropy value for each feature dimension separately to obtain the uncertainty distribution of all sensor parameters.

Combining the calculated entropy values with the self-attention mechanism, we construct an entropy adjustment coefficient to correct the attention weights and realize dual control of feature importance and data uncertainty. The entropy adjustment coefficient $\omega_{e}$ is calculated as:

(3) $$\omega_{e}=\frac{1}{1+\exp(H\left(\omega \right)-\bar{H})}$$

Where $\bar{H}$ represents the average entropy of all feature dimensions. Features with high entropy (strong uncertainty) will get a smaller $\omega_{e}$ to suppress noise interference; features with low entropy (rich valid LOCA characteristic information) will get a larger $\omega_{e}$ to strengthen feature expression. The final feature weight is the product of the self-attention weight and the entropy adjustment coefficient:

(4) $$W_{\mathrm{final}}=W_{attn}\cdot \omega_{e}$$

This strategy breaks the limitation of single attention weighting in traditional models and realizes uncertainty-aware feature optimization.

To further constrain the model from over-learning high-uncertainty noisy features during training, we construct an entropy-regularized loss function. Taking mean absolute error (MAE) as the basic regression loss, the complete loss function is:

(5) $$L_{\mathrm{total}}=L_{MAE}+\lambda \cdot \frac{1}{D}{\sum_{d=1}^{D}H_{d}\cdot \left\|w_{d}\right\|}_{2}$$

Where $L_{MAE}$ is basic mean absolute error loss for break size prediction; λ is regularization coefficient (empirically set to 0.01 in this study); *D* is total number of feature dimensions (*D* = 93); $H_{d}$ is information entropy of the *d*-th feature; $w_{d}$ is network weight vector corresponding to the *d*-th feature; ${\left\|\cdot \right\|}_{2}$ is *L*2 norm.

The entropy regularization term penalizes the network weights corresponding to high-entropy noisy features, which effectively improves the model’s robustness under complex LOCA transient conditions.

### 3.5. Overall Network Topology and Layer Configuration

This section describes the complete topology, layer sequence, and detailed parameter settings of the proposed entropy-driven CNN-LSTM-Attention hybrid model. The CNN module consists of two convolutional layers and two max-pooling layers arranged alternately. The first convolutional layer adopts 32 convolution filters with a kernel size of 3 and uses the ReLU activation function, followed by a max-pooling layer with a pooling size of 2. The second convolutional layer is equipped with 64 convolution filters and a kernel size of 3, also using the ReLU activation function, and is followed by another max-pooling layer with a pooling size of 2. This module is responsible for extracting spatial coupling features from multi-sensor time series data. After the CNN module, one independent LSTM layer is deployed to capture long-term temporal dependencies of accident transient data. This LSTM layer is configured with 128 hidden units. The self-attention layer is placed directly after the LSTM layer, which means the output features of the LSTM layer are taken as the input of the self-attention layer. The self-attention layer works together with the entropy-driven weighting module to correct feature weights and suppress high-uncertainty noise information. Two fully connected layers are set at the end of the network for regression prediction of break size. The first fully connected layer contains 64 neurons and applies the ReLU activation function. The second fully connected layer serves as the output layer with only 1 neuron and no additional activation function, which outputs the final predicted break size value. All above layer configurations remain consistent throughout all comparison experiments.

## 4. Experimental Results

Accurately assessing the size of the LOCA breach is central to evaluating algorithm performance. This paper employs five statistical metrics, namely Mean Absolute Error (MAE), Mean Relative Error (MRE), Mean Absolute Percentage Error (MAPE), Mean Square Error (MSE), and Correlation Coefficient (CC), to quantify prediction accuracy and comprehensively evaluate performance, as shown in Equations (1)–(4) [28]. MAE reflects the average level of absolute deviation between predicted and actual values; its units are consistent with the original data, but it has limitations in that it loses directional information and lacks sensitivity to scale. MRE is the average of relative deviations; after normalization, it allows for comparison of prediction accuracy across data of different magnitudes, but it fails to compute accurately when actual values approach zero; MAPE avoids the issue of positive and negative errors canceling each other out by taking the absolute value of the relative error and then averaging it; the smaller the value, the higher the accuracy. MSE is the average of the sum of squared errors; the larger the value, the lower the accuracy, and it is a commonly used accuracy metric in regression analysis and least squares methods. CC is used to characterize the correlation relationship and degree between actual and predicted values, with a range of (−1, 1); the closer the absolute value is to 1, the higher the correlation. In LOCA breach size prediction, MAE and MRE are used to reflect the error between predicted and actual values, while the prediction evaluation module employs MSE, MAPE, and CC. Among these, CC is independent of the data scale and can serve as an absolute evaluation metric for different prediction objects, whereas MSE and MAPE are dependent on the data scale and are primarily used to compare the performance of different prediction methods for the same prediction object. Key hyperparameters, including the historical time step T, batch size, and initial learning rate, are determined by grid search combined with the characteristics of LOCA time-series data and practical experience to balance model performance and computational efficiency.

(6) $$\mathrm{MAE}=\frac{1}{n}\sum_{i=1}^{n}\left|{\hat{x}}_{i}-x_{i}\right|$$

(7) $$\mathrm{MRE}=\frac{1}{n}\sum_{i=1}^{n}\frac{{\hat{x}}_{i}-x_{i}}{x_{i}}$$

(8) $$\mathrm{MAPE}=\frac{1}{n}\sum_{i=1}^{n}\left|{\hat{e}}_{i}\right|=\frac{1}{n}\sum_{i=1}^{n}\left|\frac{{\hat{x}}_{i}-x_{i}}{x_{i}}\right|$$

(9) $$\mathrm{CC}=\frac{n\sum_{i=1}^{n}\left({\hat{x}}_{i}\cdot x_{i}\right)-\left(\sum_{i=1}^{n}{\hat{x}}_{i}\right)\left(\sum_{i=1}^{n}x_{i}\right)}{\sqrt{n\left(\sum_{i=1}^{n}{\hat{x}}_{i}^{2}\right)-{\left(\sum_{i=1}^{n}x_{i}\right)}^{2}}\sqrt{n\left(\sum_{i=1}^{n}x_{i}^{2}\right)-{\left(\sum_{i=1}^{n}{\hat{x}}_{i}\right)}^{2}}}$$

where $x_{i}$ represents the actual value, ${\hat{x}}_{i}$ stands for the predicted value, and *n* is the total sample size.

**Figure 7:**
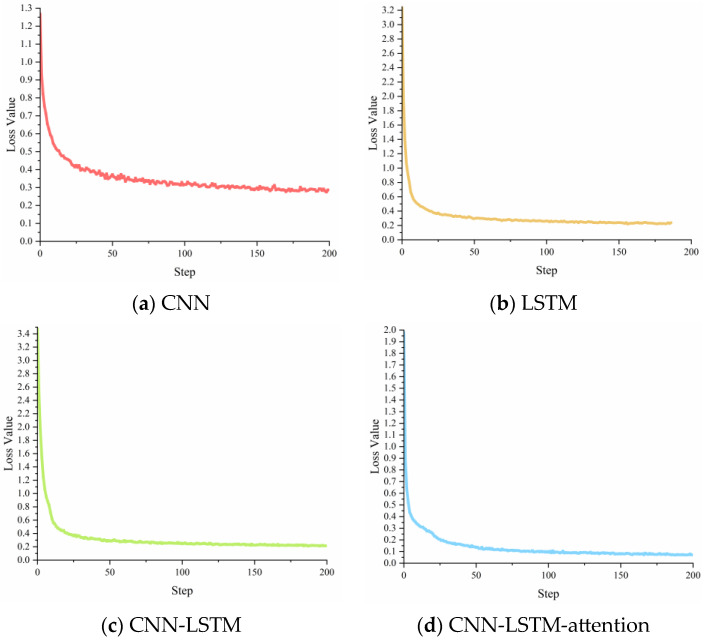
Model Training Loss Function.

The training loss function is used to quantify the error between the model’s predictions on the training data and the actual results under the current parameters. Figure 7 shows the training loss curves for four models: CNN, LSTM, CNN-LSTM, and CNN-LSTM-Attention. While all models exhibit good convergence performance, there are significant differences in their convergence behavior and training stability; the curves intuitively reflect the dynamic characteristics of the optimization process for each model.

As illustrated in Figure 1, for the CNN model, the loss rapidly decreased to around 0.4 during the first 40 iterations before entering a plateau phase, eventually stabilizing near 0.25. While the convergence rate was fast, further optimization was limited in the later stages, reflecting the insufficient ability of a single convolutional network to model temporal dependencies and its difficulty in capturing long-range dynamic correlations among accident parameters. The LSTM model exhibited significant fluctuations during convergence, with a rebound in loss occurring between iterations 80 and 120, eventually stabilizing around 0.3. This phenomenon is related to the training instability of recurrent neural networks; while they can capture temporal features, they are sensitive to local abrupt signals such as sudden pressure drops at the breach, which can easily cause oscillations in the loss function. The loss of the CNN-LSTM hybrid model rapidly decreased to 0.3 within the first 60 iterations and subsequently converged steadily to 0.2–0.25. Its overall stability outperforms that of standalone models, as the local feature extraction capability of the convolutional layers and the temporal dependency capture capability of the LSTM form an initial synergy. However, the rate of loss reduction slowed after 100 iterations, indicating that the model’s ability to focus on key features still has room for improvement. The CNN-LSTM-Attention model exhibited the smoothest convergence process. After 120 iterations, the loss dropped below 0.1 and eventually stabilized around 0.08, with the minimum loss reduced by more than 60% compared to other models. The introduction of the attention mechanism allows the model to continue optimizing even in the later stages of training. It dynamically adjusts feature weights, amplifying the influence of key parameters related to breach scenarios, such as pressure drop rates and sudden changes in coolant flow, while reducing interference from redundant information, thereby achieving more precise gradient descent.

As shown in Table 1, the CNN-LSTM-Attention hybrid model demonstrates a significant advantage in terms of absolute error performance, with an MAE of 0.096311. Compared to CNN (0.282422), LSTM (0.326316), and CNN-LSTM (0.270665), representing reductions of 66.2%, 70.5%, and 64.4%, respectively, demonstrating a substantial improvement in the ability to control absolute deviations in break size. Particularly in the detection of breaks with areas < 1 cm^2^, the model’s MAE is consistently maintained within 0.05 cm^2^, fully meeting the high-precision requirements for detecting minute leaks in engineering applications. In relative error evaluation, the fusion model also performed excellently, with an MRE of +0.11%, which is near zero, indicating no systematic prediction bias; in contrast, the CNN, LSTM, and CNN-LSTM models exhibited positive, negative, and significant negative biases of +2.96%, −0.78%, and −6.95%, respectively, all showing varying degrees of prediction offset. Under the MAPE metric, the fusion model achieved 2.81%, significantly outperforming the CNN’s 6.72%, the LSTM’s 8.24%, and the CNN-LSTM’s 14.56%; for extreme breach conditions, this model can stably maintain MAPE within 3%, effectively resolving the issue of excessive prediction bias in traditional models under such scenarios. In terms of volatility, the fusion model’s MSE is 0.085162, the lowest among all models, representing a 38.1% reduction compared to the best-performing baseline model, CNN-LSTM (0.137684), and demonstrating superior stability in prediction results. Furthermore, the correlation coefficient (CC) between the fusion model’s predicted values and the actual values reaches 0.994902, approaching the ideal value of 1, indicating the strongest linear correlation between the two. This demonstrates that the model can more effectively capture the complex nonlinear mapping relationship between break size and monitoring parameters.

**Figure 8:**
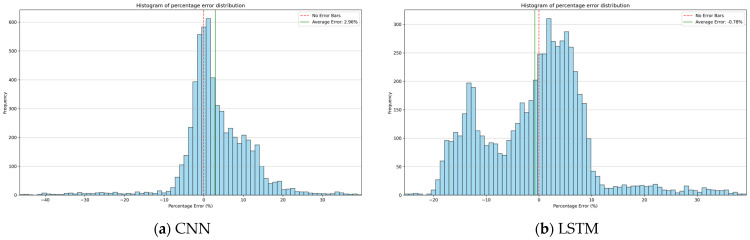

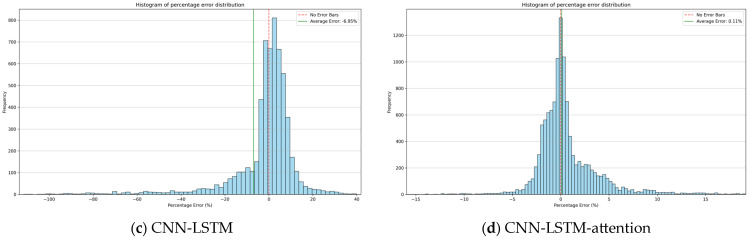
Model Error Percentage Histogram.

The histogram of percentage error distribution reveals, from a statistical perspective, the characteristics of the prediction error distribution for each model under different operating conditions (Figure 8). The CNN model exhibits a right-skewed error distribution, primarily concentrated in the −10% to 20% range, with an average error of +2.96%. Samples in the 10–20% range account for approximately 35%, indicating a systematic overestimation of break in the 1–5 cm^2^ range; The LSTM model’s error distribution extends from −20% to 30%, with an average error of −0.78%. Samples in the −10% to 0% range account for over 60%. Due to overfitting of temporal dependencies, predictions for breaks larger than 5 cm^2^ are generally underestimated. The CNN-LSTM model exhibits significant dispersion in its error distribution, ranging from −100% to 40%, with an abnormal peak in the −20% to −60% range (accounting for approximately 25% of the samples); with an average error of −6.95%. Due to insufficient capture of multi-parameter coupled features, it is prone to extreme deviations under complex operating conditions where the breach location and initial power interact. In contrast, the CNN-LSTM-Attention model exhibits a narrow-peak error distribution, with over 90% of samples concentrated within the −5% to 5% range and an average error of 0.11%. The distribution is nearly unbiased and approximates a normal distribution. The attention mechanism effectively suppresses interference from irrelevant features and reduces the probability of extreme errors by dynamically focusing on key parameters such as the rate of pressure change in the loop 10 s after a rupture. The model demonstrates excellent predictive stability across various operating conditions. From the perspective of nuclear thermal hydraulics, small-break LOCA produces faint transient signals easily masked by noise; the attention mechanism captures subtle thermal-hydraulic variations and enhances identification accuracy. Meanwhile, the model achieves fast inference speed, satisfactory real-time performance, good noise robustness, and acceptable few-shot generalization ability for engineering applications.

**Figure 9:**
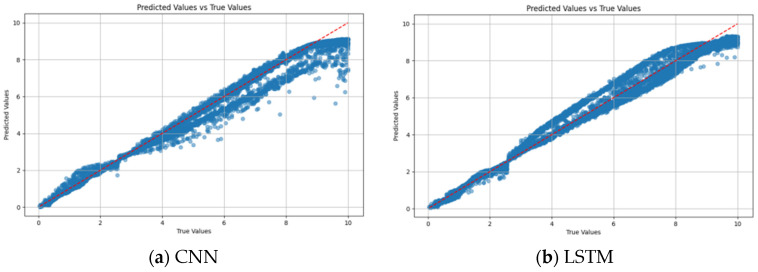

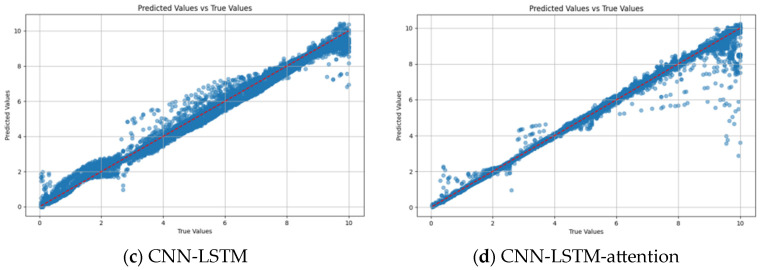
Scatter Plot of Models’ Predictions and True Values.

Figure 9 shows a scatter plot of the actual and predicted values for the four models, providing a visual representation of the consistency of the model evaluations. Ideally, the data points should be closely clustered around the 45° reference line. The data points for the CNN model show significant dispersion in the region where the breach is <1 cm^2^, deviating considerably from the reference line; over 30% of the samples fall outside the ±15% error band, indicating insufficient ability to identify faint leakage signals from small breaches; The LSTM model’s data points deviate markedly in the >8 cm^2^ rupture area and systematically shift below the reference line (averaging 12.7% lower), indicating a deficiency in modeling the strong transient characteristics of large ruptures; The CNN-LSTM model’s point cloud generally aligns more closely with the reference line, showing significant improvement over the standalone base model. However, it suffers from localized failures, exhibiting significant fluctuations in the 2–4 cm^2^ range. The point cloud displays a “butterfly-shaped” distribution with a lateral spread of up to ±20%, indicating insufficient stability in capturing the parameter coupling characteristics of leaks in this range. In contrast, the point cloud of the CNN-LSTM-Attention model almost perfectly aligns with the 45° reference line, with 98.2% of samples falling within a ±5% error margin, maintaining high consistency across the entire breach size range of 0.05–10 cm^2^. This confirms the reinforcing role of the attention mechanism’s key features, which effectively enhance the model’s evaluation accuracy across the full range of gaps by focusing on core parameters strongly correlated with gap size, such as the rate of change in the stabilizer water level and the decay rate of coolant flow.

Based on the above experimental conclusions, an analysis across four dimensions, training stability, quantitative metric performance, error distribution characteristics, and evaluation consistency, reveals that all four models can effectively assess SMR-LOCA breach sizes. However, the CNN-LSTM-Attention model demonstrates comprehensive performance advantages: Its training convergence process is stable, all quantitative evaluation metrics significantly outperform those of other models, the error distribution is concentrated and close to unbiased, and it demonstrates excellent consistency in breach size assessment across the entire range. The model’s superior performance stems from the synergistic interaction and efficient integration of its three major components: the CNN layer precisely extracts local spatial coupling features among multiple parameters, such as the transient interdependence between the pressurizer water level and primary loop pressure; the LSTM layer effectively captures the dynamic evolution of features over time, such as the decay trend of coolant flow within 300 s after a breach; and the attention mechanism dynamically allocates feature weights to focus on critical periods and core parameters like the rate of pressure drop, thereby forming a triple feature extraction mechanism of “spatial feature extraction-temporal evolution capture-key information focus.” This mechanism effectively overcomes the modeling bottlenecks of traditional models regarding highly nonlinear and multi-parameter coupled characteristics, providing a high-precision technical solution for the intelligent diagnosis of LOCA accidents in SMRs.

## 5. Conclusions

Accurate break size assessment under loss-of-coolant accidents (LOCAs) is essential to the safety mitigation and emergency response of small modular reactors (SMRs). The strong spatiotemporal coupling, rapid transient evolution, and high uncertainty characteristics of SMR-LOCA lead to significant challenges for traditional assessment methods, which suffer from low accuracy and poor stability. To tackle these issues, this study developed an entropy-driven intelligent diagnosis framework based on a CNN-LSTM-Attention hybrid model for high-precision break size evaluation. A high-quality time-series dataset covering break sizes from 0.05 cm^2^ to 10 cm^2^ was established using the PCTRAN/SMART platform. The proposed model integrates the spatial feature extraction capability of CNN, the temporal dependency learning ability of LSTM, and the adaptive key information focusing of the attention mechanism, forming a unified spatiotemporal learning architecture that effectively suppresses uncertainty and improves prediction robustness.

Experimental results demonstrate that the CNN-LSTM-Attention model outperforms standalone CNN, LSTM, and CNN-LSTM models in all metrics. It achieves a mean absolute error (MAE) of 0.096311, with error reduction exceeding 64.4% relative to baseline models. Over 90% of prediction errors are controlled within ±5%, and the correlation coefficient reaches 0.994902, indicating excellent consistency between predicted and actual break sizes. The entropy-driven weighting strategy enhances the model’s sensitivity to critical transient features and reduces the impact of redundant information, enabling stable and high-precision full-range assessment across all break sizes. Distinct from the existing CNN-LSTM-Attention hybrid structures widely applied in nuclear accident diagnosis, the proposed entropy-driven framework is the major advancement of this research. By quantifying data uncertainty via information entropy and combining entropy-adaptive weighting with loss regularization, this method effectively solves the problem that traditional models cannot distinguish valid signals from uncertain noise under SMR-LOCA conditions. This is the exclusive contribution of the entropy-driven design in this study.

This work provides an intelligent tool for uncertainty quantification, accident diagnosis, and safety assessment of SMRs. Although the current research is based on the simulation data of PCTRAN/SMART, this simulation platform has passed the certification of multiple nuclear safety institutions and can accurately reproduce the actual operating and accident characteristics of the SMART reactor, which ensures the validity of the model verification results. Different from previous SMR-LOCA diagnosis methods that only focus on model structure optimization, this work takes data uncertainty as the core optimization direction and forms a complete set of spatiotemporal feature learning + entropy constraint technical scheme. In future research, the model will be further validated against experimental data and multiple thermal-hydraulic simulation platforms, extended to multitype and complex accident conditions, and optimized toward real-time engineering applications in nuclear power plant monitoring and emergency operation support.

## Figures and Tables

**Table 1:** Evaluation Metrics Table for CNN, LSTM, CNN-LSTM, and CNN-LSTM-Attention Models.

Models	MAE	MRE	MAPE	MSE	CC
CNN	0.282422	2.96%	6.72%	0.217054	0.990032
LSTM	0.326316	−0.78%	8.24%	0.181952	0.989213
CNN-LSTM	0.270665	−6.95%	14.56%	0.137684	0.992443
CNN-LSTM-Attention	0.096311	0.11%	2.81%	0.085162	0.994902

## Data Availability

Data are available on request due to restrictions.
